# Evaluation of Phytochemical Screening, Pigment Content, In Vitro Antioxidant, Antibacterial Potential and GC-MS Metabolite Profiling of Green Seaweed *Caulerpa racemosa*

**DOI:** 10.3390/md21050278

**Published:** 2023-04-28

**Authors:** Sivagaami Palaniyappan, Arun Sridhar, Zulhisyam Abdul Kari, Guillermo Téllez-Isaías, Thirumurugan Ramasamy

**Affiliations:** 1 Laboratory of Aquabiotics/Nanoscience, Department of Animal Science, School of Life Sciences, Bharathidasan University, Tiruchirappalli 620 024, Tamil Nadu, India; sivapls95@gmail.com; 2 Immunology-Vaccinology, Department of Infectious and Parasitic Diseases, Fundamental and Applied Research for Animals & Health (FARAH), Faculty of Veterinary Medicine, University of Liège, Liège 4000, Belgium; arun.sridhar@uliege.be; 3 Department of Agricultural Sciences, Faculty of Agro-Based Industry, Universiti Malaysia Kelantan, Jeli Campus, Jeli 17600, Malaysia; zulhisyam.a@umk.edu.my; 4 Advanced Livestock and Aquaculture Research Group, Faculty of Agro-Based Industry, Universiti Malaysia Kelantan, Jeli Campus, Jeli 17600, Malaysia; 5 Department of Poultry Science, University of Arkansas, Fayetteville, AR 72701, USA; gtellez@uark.edu

**Keywords:** antibacterial activity, antioxidant activity, bioactive compounds, GC-MS, seaweed

## Abstract

Exploration of seaweeds to unravel their bioactive metabolites from the perspective of wider applications gained substantial importance. The present study was performed to investigate the total phenolic, flavonoid, tannin content, antioxidant activity and antibacterial potential of various solvent extracts of green seaweed *Caulerpa racemosa*. The methanolic extract showed higher phenolic (11.99 ± 0.48 mg gallic acid equivalents/g), tannin (18.59 ± 0.54 mg tannic acid equivalents/g) and flavonoid (33.17 ± 0.76 mg quercetin equivalents/g) content than other extracts. Antioxidant activity was determined by using 2,2-diphenylpicrylhydrazyl (DPPH) and 2,2′-azino-bis(3-ethylbenzothiazoline-6-sulfonic acid) (ABTS) assay with different concentrations of *C. racemosa* extracts. The methanolic extract showed higher scavenging potential in both the DPPH and ABTS activity with the inhibition value of 54.21 ± 1.39% and 76.62 ± 1.08%, respectively. Bioactive profiling was also identified by using Gas chromatography-mass spectrometry (GC-MS) and Fourier transform infrared (FT-IR) techniques. These studies revealed the presence of valuable bioactive compounds in *C. racemosa* extracts and these compounds might be responsible for antimicrobial, antioxidant, anticancer and anti-mutagenic properties. Major compounds identified in GC-MS were 3,7,11,15-Tetramethyl-2-hexadecen-1-ol, 3-hexadecene and Phthalic acid. In terms of antibacterial activity, *C. racemosa* has promising antibacterial potential against aquatic pathogens *Aeromonas hydrophila, Aeromonas veronii* and *Aeromonas salmonicida*. Further evaluation studies focusing aquatic related aspects would reveal the novel bioproperties and applications of *C. racemosa*.

## 1. Introduction

The marine environment is rich in biodiversity with numerous potentials and contains bioactive compounds of unique structural and physical properties that are inimitable to the natural molecules derived from terrestrial sources [1]. Macroalgae collectively known as seaweed are an integral part of the marine ecosystem. Seaweeds are considered as non- flowering, photosynthetic plant-like organisms which play a vital role as (i) primary producers in the marine niche; (ii) food sources for herbivorous organisms and (iii) habitats for many microorganisms [2]. Seaweed consumption as food or medicine was already recorded since ancient times and now, it became a popular ingredient in the preparation of food and beverages [3]. In Western countries, macroalgae became prevalent foods latterly due to the presence of many beneficial properties. Every year, 20 million tons of seaweed were harvested and half of them were intended for human consumption [4,5]. In addition, compounds and metabolites present in seaweeds are in high demand with extensive applications in cosmetics and pharmaceutical industries [6]. Seaweeds contain high-quality proteins, dietary fibers, polysaccharides, macro and micronutrients, vitamins, minerals, fatty acids and phytochemical constituents/bioactive compounds which possess a wide spectrum of activities [7,8,9]. The seaweed shows extensive species diversity distribution and abundance. Depending on species, season, temperature and geographic locations, the biochemical contents may vary, and these factors influence their minerals and elements. Furthermore, seaweeds are the only source of compounds such as agar, algin and carrageenan, which are used as gelling and stabilizing agents [10]. Seaweeds provide cobalamin (vitamin B_12_) which is not synthesized or acquired by higher plants [11]. Seaweeds are also used as a productive source of biomass for its simple depolymerization ability owing to the absence of hard lignocellulose [9].

Depending on pigments, seaweeds are classified into three groups, such as red (Rhodophyceae), brown (Phaeophyceae) and green algae (Chlorophyceae) [12]. Each macroalgae has unique biological properties with a wide range of applications. Macroalgae were utilized in various fields based on their characteristics features and chemical compositions. Altogether, these macroalgae provide many socio-economic values. In recent years, macroalgae garnered huge interest due to their potential use in feed, pharmaceutics and an increased application in health-promoting functional foods. Proportionately, the aquaculture farming of green seaweeds was expanded over the last decade for commercialization [13]. Green algae of the genus *Caulerpa*, family *Caulerpaceae*, are distributed worldwide in shallow water subtropical and tropical marine habitats and they are contemplated as better alternative food with therapeutic properties [14]. The species *Caulerpa racemosa* commonly referred to as “sea grapes” is one of the dominant edible marine green seaweeds on the Indian coastline and a good source of magnesium, iron and calcium. It is consumed in raw or cooked forms across the Indo-pacific regions [15]. It displays invasive behavior and has the tendency to propagate clonally by fragmentation and become a major feeding habit of demersal species [16]. The *C. racemosa* contains phytoconstituents (ceramides, sesquiterpenes etc), amino acids (alanine, phenylalanine, glutamic acid, glycine, serine, isoleucine, lysine, aspartic acid, leucine and valine) and peptides [17]. *C. racemosa* is known for its polyunsaturated fatty acids (PUFA), secondary metabolites which are responsible for its antibacterial, anticancer, antinociceptive, antimutagenic, anti-inflammatory and cytotoxic properties. The antioxidant capacity of *C. racemosa* highlights its potential utilization as nutraceuticals. *C. racemosa* was shown to have anti-aging and anti-obesity properties by altering glucose and lipid profiles [18]. Recent evidence suggested that *C. racemosa* could be used as a functional food with beneficial applications in human health [19].

In particular, specific bisindole alkaloid compounds such as caulerpin, caulersin and caulerpenyne are rich in *C. racemosa.* They exert a wide array of bioactivities and are highly desirable in multi-industrial applications [20]. Racemosin A & B and caulerprenylols A & B isolated from *C. racemosa* exhibited neuroprotective and antifungal activity, respectively [21]. The extracts of *C. racemosa* inhibit the growth of bacterial pathogens which cause infections in humans, plants and animals. It was reported that the bacteria associated with *C. racemosa* have the capacity to inactivate the pathogens causing disease in *Gracillaria verrucosa* species. Thus, co-culturing of *C. racemosa* and *G. verrucosa* may benefit to meet out the demand of *Gracillaria* species for export by reducing the disease occurrence [2]. The polysaccharides of *C. racemosa* have immunomodulatory or immunostimulatory effects that contribute in a great manner for the pharmaceutical industries to treat different types of diseases [22]. Moreover, the supplementation of *C. racemosa* to the *Vibrio parahaemolyticus* infected white shrimp (*Litopenaeus vannamei*) can increase the survival rate by increasing the macrophage activity with the help of sulfate polysaccharides [23]. Although much research endeavors were studied in *C. racemosa*, there are still some gaps to fill the existing knowledge concerning its bioactive constituents. In order to investigate the medicinal properties of *C. racemosa*, it is necessary to study the active biomolecules and its interactions [19]. Therefore, the present investigation was carried out to study the metabolites of different solvent extracts of *C. racemosa* through GC-MS profiling and in vitro studies that will provide important biomolecules insights and exploit its further potential in the aspects of human and animal health.

## 2. Results and Discussion

### 2.1. Pigments Determination

**Figure 1:**
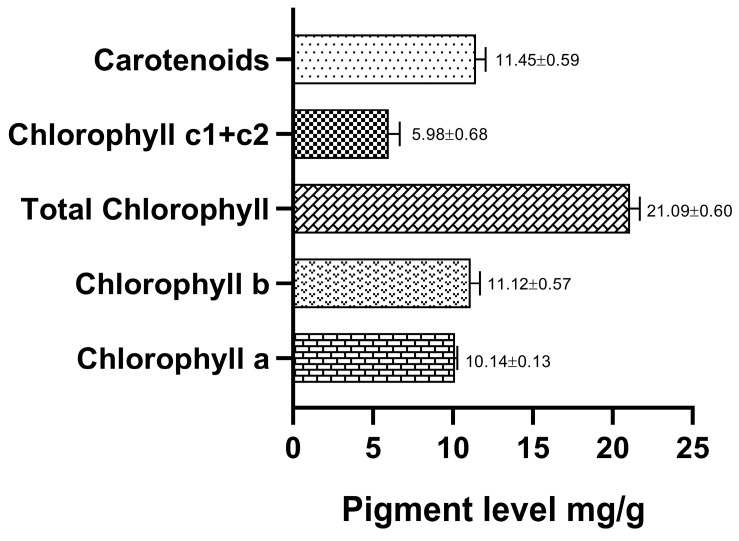
Pigments content of collected green seaweed *Caulerpa racemosa*.

Pigments are used to absorb the light for photosynthesis in seaweeds. They can act as an antioxidant by removing free radicals and preventing oxidative damage [24,25]. Chlorophyll and carotenoids (carotenes and xanthophylls) are the major pigments present in green seaweeds [26]. In this study, chlorophyll a, chlorophyll b, chlorophyll c1+c2, total chlorophyll and carotenoids were evaluated and results are manifested in Figure 1. Chlorophyll c is a pigment-protein light-harvesting complex which allow light to penetrate underwater habitats due to its spectroscopic properties and structures [27]. Verma et al. [28] reported that *Caulerpa vervelansis* possess higher chlorophyll a pigment. These pigments in seaweeds contain various health benefits.

Provitamin-A carotenoid, and β-carotene are a significant source of vitamins. Xanthophyll pigments efficiently absorb the blue light and impede the formation of reactive oxygen species in the photoreceptors that helps to defend from light-induced oxidative damage in the retinal pigment epithelial cells [29,30]. These macroalgae pigments show diverse activities such as antioxidant activity, neuroprotective effects and cardiovascular protection [31].

### 2.2. Biochemical Constituents Analysis

The proximate composition of *C. racemosa* (CR) powder is presented in Table 1. Proximate composition analysis is very crucial for the assessment of nutritional value of macronutrients and could be used to formulate feed. Dried powder of *C. racemosa* contained 7.04% of moisture, 12.64% of crude protein, 2.85% of crude fibre, 1.8% of ether extract, 48.41% of total ash and 2089 Kcal/kg of gross energy. Our results were in line with Hao et al. [32] in *C. racemosa var peltata*. Regal et al. [33] evaluated the proximate composition of seaweed *Asparagopsis taxiformis* and reported the similar level of ash content (47.3 to 48.7%).

### 2.3. Preliminary Phytochemical Analysis 

Seaweeds contain unique phytochemicals that are associated with a plenty of biological activities and they are believed to hold various health benefits [34]. The phytochemicals include tannins, flavonoids, saponins, phytosterol, terpenoids, phenol, phenolic flavonoids, alkaloids and steroids of various extracts of *C. racemosa* were screened and depicted in Table 2. All the phytochemicals screened in this study were present in polar solvent (methanol and ethanol) extracts. Terpenoids, steroids, phytosterols, tannins and flavonoids were present in ethyl acetate extract. Acetone extract showed the absence of saponins and alkaloids. Petroleum ether extract exhibited the presence of tannins. Terpenoids and tannins were present in the hexane extract of *C. racemosa*.

According to Nagaraj and Osborne. [10], the methanolic extract of *C. racemosa* demonstrated the presence of saponins, alkaloids and terpenoids. These secondary metabolites have numerous therapeutic benefits and are used tremendously in the drug and pharmaceutical industry. Tannin and saponins are the excellent anti-microbial agents, while flavonoids and polyphenols are antioxidant agents. Flavonoids are water-soluble antioxidants that can scavenge free radicals. Flavonoids in human diet may prevent menopausal symptoms and reduce cancers [35,36]. Alkaloids were nitrogenous compound that contains anti-inflammatory, anti-fungal and antibacterial activities [37]. The macroalgae genus *Caulerpa* contains a high amount of indolic alkaloid compound caulerpin, which was reported to possess anti-inflammatory activity. Caulerpin was reported in various species of Caulerpa genus of green seaweeds such as *C. racemosa, C. lentillifera, C. peltata, C. paspaloides, C. cupressoides, C. sertularioides, C. prolifera* and *C. mexicana* [38].

### 2.4. FT-IR Analysis

**Figure 2:**
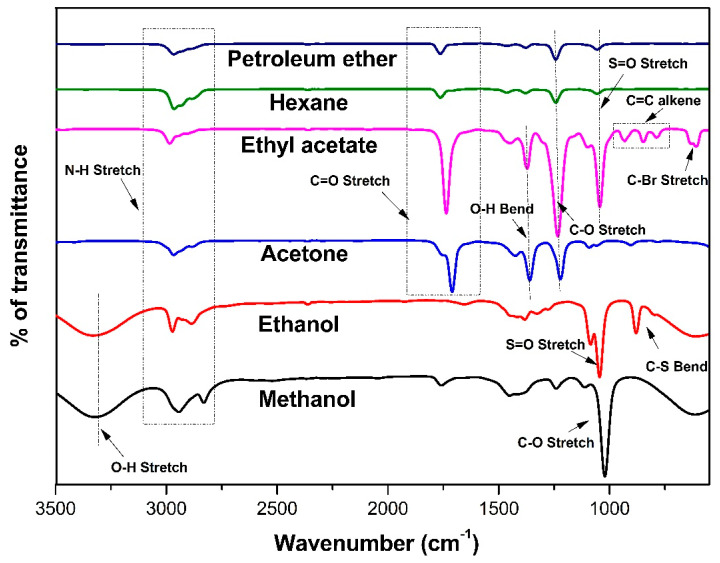
FT-IR spectrum of various extracts of *Caulerpa racemosa*.

Based on the wavelength and intensity of the absorption bands of different molecular groups, FT-IR spectroscopy can reveal the presence of chemical components. This method is extensively used in food authentication and efficient in capturing the entire composition of chemical compounds [39,40]. Functional groups were detected by the infrared radiation ranges from 4000 to 500 cm^−1^ (Figure 2). Based on the FT-IR results obtained in this study, methanolic and ethanolic extracts of CR showed the absorption band at 3325 cm^−1^ (OH stretch alcohol). All extracts of CR showed a strong peak between 2972.73 and 2943.8 cm^−1^ (NH stretch amine salt).

C=O stretch indicated the presence of aliphatic ketones in ethyl acetate, acetone, hexane and petroleum ether extract of *C. racemosa* at the band range of 1710.55, 1736.58, 1763.58 and 1763.85 cm^−1^, respectively. The OH bend represented the carboxylic group at the peak of 1449.24 cm^−1^ in ethyl acetate extract and 1425.14 cm^−1^ in acetone extract. The peaks were observed around 1242.9 to 1221.68 cm^−1^ in hexane, petroleum ether, ethyl acetate and acetone extract of *C. racemosa*, indicating the presence of alkyl ether. A strong peak at 1021.12 cm^−1^ (C-O) in the methanol extract of CR indicated the presence of ether [41]. In the ethanol extract of CR, the medium peak at 879.38 cm^−1^ represented the C-S bend [42]. Halo compounds were observed at the peak range of 609.39 cm^−1^ in ethyl acetate extract of CR. The FT-IR results revealed the presence of various bioactive molecules in the extracts of *C. racemosa*. These compounds are responsible for its anti-bacterial, antioxidant and other medicinal properties.

### 2.5. GC-MS Analysis

**Figure 3:**
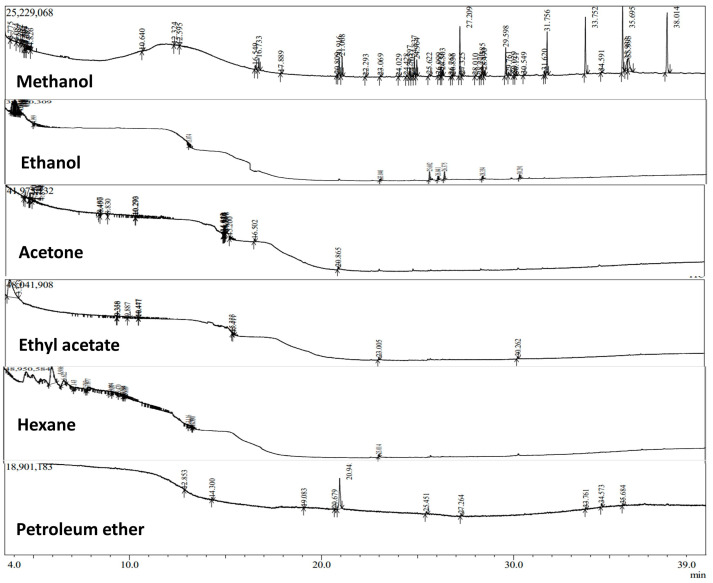
GC-MS chromatogram of various extracts of *Caulerpa racemosa*.

The GC-MS chromatogram and detected compounds of *C. racemosa* extracts are given in Figure 3 and Table 3. In total, 74 compounds were identified from various extracts of *C. racemosa*. The highest number of compounds were detected in the methanol extract (29 compounds) and the lowest number of compounds were detected in hexane (7 compounds) extract of *C. racemosa*. In the present study, all the CR extracts contained bioactive compounds that exhibit antimicrobial, antioxidant, anticancer and anti-mutagenic properties.

The major metabolite identified was carboxylic acid and it is an important antioxidant [43]. 3-hexadecene shows numerous medicinal properties to cure cancer, inflammatory diseases and diabetes [44]. Phthalic acid has antibacterial and antioxidant properties. Phthalic acid inhibits the oxidation by stabilizing the phenoxyl radicals [45]. Methyl glycolate is a potential antioxidant reported by Shah et al. [20]. Tetrazole has antimicrobial property [46]. 8,11,14-docosatrienoic acid methyl ester is one of the (n-6 fatty acids) polyunsaturated fatty acids [47,48]. 3,7,11,15-Tetramethyl-2-hexadecen-1-ol displays antimicrobial activity [49]. The GC–MS results from the various extracts of CR confirmed that they all possessed numerous beneficial compounds.

### 2.6. Total Phenolic Content

**Figure 4:**
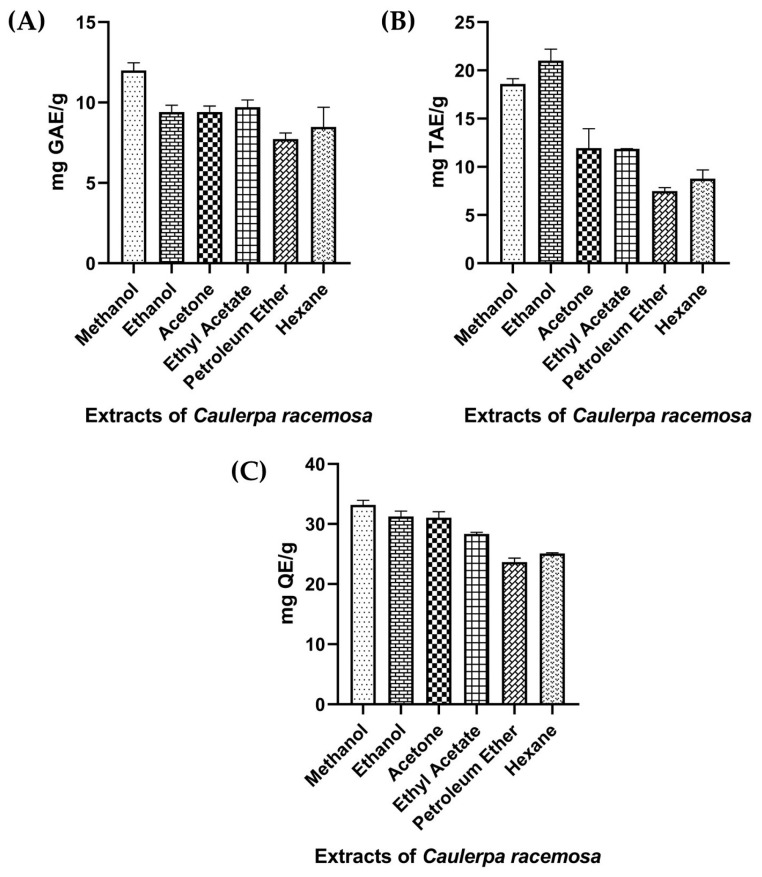
Quantitative analysis of (**A**) total phenolic content, (**B**) total tannin content and (**C**) total flavonoid content of *Caulerpa racemosa* extracts.

In higher plants, phenolic compounds in the secondary metabolite forms are prevalent bioactive compounds [50]. In recent years, bioactive polyphenols received importance due to their protection efficiency against oxidative stress, which is responsible for many diseases including aging, cancer and congestive heart failure [51]. In seaweeds, phenolic compound production may differ based on varying environmental factors such as salinity, herbivory pressure, nutrients, UV radiation, etc. [52,53]. Results are shown in Figure 4A. In this study, the highest phenolic content was estimated in methanol extract of *C. racemosa* (11.99 ± 0.48 mg GAE/g) followed by ethanol (9.70 ± 0.45 mg GAE/g), acetone (9.40 ± 0.42 mg GAE/g), ethyl acetate (9.40 ± 0.38 mg GAE/g), hexane (8.48 ± 1.23 mg GAE/g) and petroleum ether (7.73 ± 0.38 mg GAE/g).

Phenols are natural antioxidants, which produce OH functional groups in seaweed that inhibit oxidative stress by donating hydrogen to stabilize and prevent free radical generation. It lowers the disease risk and promotes health [20]. Vega et al. [53] reported that 2.26% of total phenol was evaluated in *C. racemosa*. According to Akbary et al. [54] polar solvent extract of brown seaweed *Stoechospermum marginatum* exhibited higher phenolic content than other solvents used. Marinho et al. [55] reported that higher phenolic content was obtained in the methanol extract than ethyl acetate extract. Rodríguez-Bernaldo de Quirós et al. [56] evaluated the phenolic compounds of brown seaweed *Sargassum pallidum* extracts using various solvents, such as 30% ethanol, 30% methanol and 70% acetone, and reported higher phenolic content in 70% acetone extract.

### 2.7. Total Tannin Content

Tannins are the kind of water-soluble polyphenols present in terrestrial plants and marine algae. They play a crucial role in vascular plants’ defense mechanism [57]. The results of tannin content in the current study are given in Figure 4B. The levels of tannin content were higher in the ethanol extract of CR (21 ± 1.21 mg TAE/g) followed by methanol (18.59 ± 0.54 mg TAE/g), acetone (11.95 ± 1.99 mg TAE/g), ethyl acetate (11.87 ± 0.023 mg TAE/g), hexane (8.77 ± 0.89 mg TAE/g) and petroleum ether (7.49 ± 0.35 mg TAE/g). Bharath et al. [58] reported that the ethanol extract of *Turbinaria ornata* (28.01 ± 0.20 mg TAE) showed higher tannin content. Consumption of tannins-containing beverages may encourage, as it is believed, to cure or prevent plenty of diseases [59]. The highest tannin content was recorded in green seaweed *C. duthieae* by Rengasamy et al. [60]. Tannin has a potential anti-inflammatory activity [61]. Tannins are also used to treat burns as it forms a protective covering by precipitating proteins of exposed tissues [62]. It is an essential compound in antimicrobial activity owing to its inactivation of membrane-bound enzyme, cell envelope transport and microbial cell adhesions [58]. Higher and lower tannin content was reported in the 70% acetone soxhlet extract of *C. peltata* and *C. latum*, respectively [57].

### 2.8. Total Flavonoid Content

Secondary metabolites, such as flavonoids, are strong antioxidants and crucial dietary supplements for humans. In *Caulerpa* spp., luteolin, apigenin, quercetin, cyanidin, malvidin, myricetin, kaempferol and quercetagetin flavonoids were detected. These metabolites demonstrated a variety of biological functions such as immune-modulation, anti-inflammatory, antioxidant and anticancer [63]. CR methanol extract of this study exhibited higher flavonoid content (33.17 ± 0.76 µg QE/g) than other solvents and lower content of flavonoid was revealed by non-polar solvent petroleum ether (23.64 ± 0.66 µg QE/g). The levels of total flavonoid contents of various CR extracts were given in Figure 4C. Sobuj et al. [64] also obtained higher flavonoid content in methanol seaweed extract of *Padina tetrastromatica* (41.77 ± 1.59 mg of Q/g) and *Gracilaria tenuistipitata* (36.17 ± 2.38 mg of Q/g). Furthermore, the present study findings are hand in hand with the results reported by Marinho et al. [55] in which the methanol extract of *Saccharina latissima* seaweed showed higher activity than ethyl acetate. Yap et al. [65] also reported higher total flavonoid content in the aqueous extract of *C. racemosa* and *C. lentillifera*. According to the report of Suraiya et al. [9] fermented seaweed *Squatina japonica* showed higher flavonoid content than unfermented seaweed *S. japonica*.

### 2.9. Antioxidant Activity

The phenolic acids and flavonoids have electron donating capacity and prevent cells from reactive oxygen species either by inhibiting or reducing free radicals. The antioxidant activity is determined by the free radical scavenging capacity or inhibition of oxidation by different biological mechanisms [19]. 

#### 2.9.1. DPPH Activity

**Figure 5:**
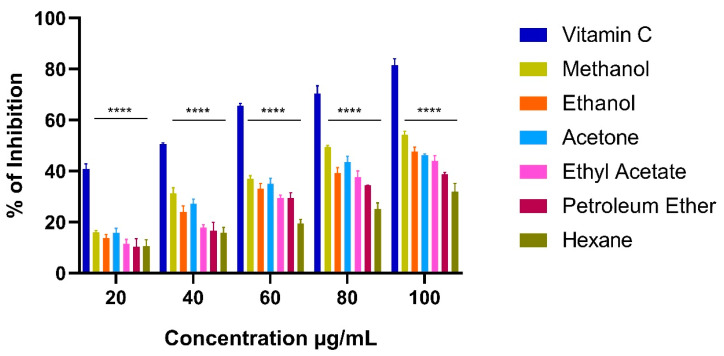
DPPH radical scavenging activity of various extracts of *Caulerpa racemosa*. Bars represent the mean ± standard deviation. Asterisks denote the significant difference between the *Caulerpa racemosa* extracts and the standard (Vitamin C).

DPPH assay is a simple and prominent method to evaluate free radical scavenging ability. The hydrogen-donating capacity of extracts was thought to be responsible for the DPPH radical scavenging activity. The antioxidant compound reacts with radical DPPH that reduces to DPPH-H, which could be observed by reduction in absorbance values [66]. Figure 5 shows the result of DPPH activity of various extracts of *C. racemosa*. It was found that the CR extracts exhibited DPPH scavenging effect in a concentration dependent manner.

In this study, all the CR extracts showed significantly lower activity than the standard in all the different concentrations. The CR extracts showed higher activity at 100 µg/mL in which the methanol extract showed activity at 54.21 ± 1.39% followed by ethanol 47.59 ± 1.78%, acetone 46.23 ± 0.46%, ethyl acetate 44.04 ± 2.01%, petroleum ether 38.79 ± 0.77% and hexane 31.86 ± 3.32%. The lowest IC_50_ value was observed in the methanol extract of CR (86.33 µg/mL) and highest IC_50_ value was obtained in the hexane extract (173.21 µg/mL) (Table 4). In our study, the total phenolic content was also higher in the methanol extract of CR, which serves as evidence of the value of phenolic compounds as antioxidants. Similar results were proclaimed by Fonseca et al. [67] in Atlantic brown seaweed species *Zonaria tournefortii* and *Cystoseira abies-marina*. Tanna et al. [63] reported that the methanol extract of *C. racemosa var. macrophysa* showed 60% of DPPH scavenging activity.

#### 2.9.2. ABTS Activity

**Figure 6:**
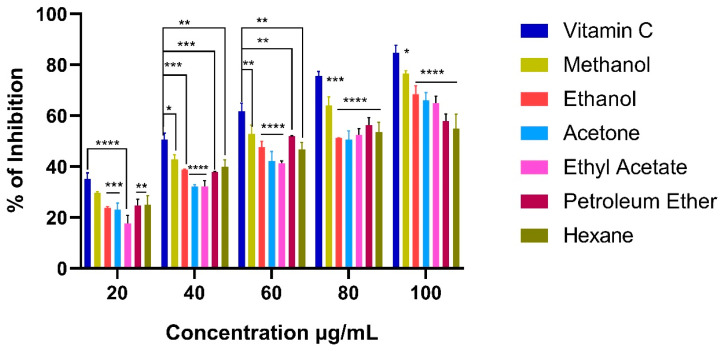
ABTS radical scavenging activity of various extracts of *Caulerpa racemosa*. Bars represent the mean ± standard deviation. Asterisk denotes the significant difference between the *Caulerpa racemosa* extracts and the standard (Vitamin C).

The decolorization of bluish-green ABTS due to polyphenolic compounds present in algal extracts was measured at 734 nm to determine the ABTS activity [68]. Similar to DPPH activity, ABTS also performed in a dose-dependent manner. The results of ABTS scavenging activity were represented in Figure 6. Highest ABTS activity was recorded in the *C. racemosa* methanol extract (76.62 ± 1.08%) followed by ethanol (68.44 ± 3.23%), acetone (66.16 ± 2.96%), ethyl acetate (64.92 ± 2.82%), petroleum ether (57.98 ± 2.69%) and hexane (54.94 ± 5.65%). Lowest IC_50_ value was expressed in methanol extract (54.51 µg/mL) and the highest IC_50_ was observed in hexane extract (76.28 µg/mL) (Table 4). According to Maheswari and Salamun. [68] the highest ABTS radical scavenging activity (96.95 ± 0.41%) was observed in *C. verticillata* than standard ascorbic acid (90.99 ± 0.30%). Mani et al. [69] evaluated the antioxidant potential of various species of tropical green seaweeds, in which *C. antennia* showed higher ABTS activity (IC_50_ 0.93 mg/mL). Subcritical water extraction of *U. lactuca* displayed a higher ABTS activity than *C. racemosa* [70].

### 2.10. Antibacterial Activity

Aquatic bacterial pathogens can cause severe economic loss in the aquaculture industry. Aeromonas is a major bacterium that causes septicaemia and ulcer in Indian major carps and other fish species. *Staphylococcus aureus*, *Klebsiella pneumoniae* and *Pseudomonas aeruginosa* are also identified as fish pathogens. *P. aeruginosa* cause red skin infection in *Oreochromis mossambicus* [71]. Divya et al. [72] stated that *P. aeruginosa* cause friable liver, gill necrosis, abdominal distension, splenomegaly and hemorrhagic septicemia in Indian major carp *Labeo rohita*. Kukułowicz et al. [73] and Sivaraman et al. [74] isolated *S. aureus* from edible fish. It affects *Oreochromis niloticus* and causes severe mortality with pathological alterations [75]. *K. pneumoniae* can also cause severe mortality in Indian major carps through causing hemorrhagic infection. The present study divulged the antibacterial potential of different extracts of *C. racemosa* against all tested aquatic bacterial pathogens. The CR methanol extract showed better activity than other extracts against all tested organisms, especially *Aeromonas veronii*. Significant variations were observed depending on solvent and pathogens when compared with control (streptomycin). The present study’s results are represented in Table 5. A higher inhibition zone was observed in methanol (27 ± 0.71 mm) and ethanol (25 ± 0.35 mm) extract of *C. racemosa* (200 µg/mL) against *A. veronii*. Petroleum ether extract (200 µg/mL) showed the lowest inhibition zone against *K. pneumoniae* (11 ± 1.41 mm). These results were similar to those obtained in the analysis of antibacterial activity of *C. racemosa* against *S. aureus* [9]. Several studies were conducted on antibacterial activity of *C. racemosa* extracts which exhibited better inhibition activity against most of the pathogenic organisms [76]. Belkacemi et al. [77] stated that methanol and hexane extract of *C. racemosa* showed inhibition zone at 10 mm and 9.33 mm, respectively, against *P. aeruginosa*. In our present study, preliminary phytochemical analysis revealed the presence of secondary metabolites such as saponins, tannins, terpenoids, etc.; these metabolites may inhibit the growth of the bacterial pathogens. Our study also disclosed the higher tannin and phenolic content in methanol extract, the same extract showed better antibacterial activity against all tested organisms. Tannin plays a major role in antimicrobial activity by inactivating membrane-bound enzymes, transport proteins and cell-to-cell adhesions [58]. Fatty acid derivatives were also identified in the GC-MS analysis, which may contribute to the antimicrobial activity of solvent extracts [78]. Talreja et al. [79] investigated the antibacterial potential of *Ulva lactuca*, and methanolic extract showed strong activity against *S. aureus*.

### 2.11. MIC Determination

Minimum inhibitory concentration (MIC) is the lowest concentration of an agent that prevents microbial growth [80]. The MIC of CR extracts was determined by the Resazurin-based 96-well plate dilution method [81]. The MIC of each CR extract was determined visually by the color change in the 96-well plate. Positive control streptomycin showed MIC at 25 µg/mL against *A. hydrophila*, *A. veronii* and *A. salmonicida;* 50 and 100 µg/mL of MIC were determined against *Staphylococcus aureus* and *Klebsiella pneumoniae*, respectively. The results are shown in Table 5. Methanol, ethanol and acetone extracts of *C. racemosa* exhibited similar MIC values (100 µg/mL) against all the tested Aeromonas strains. Hexane and petroleum ether extract showed the MIC value at 400 µg/mL against *P. aeruginosa, S. aureus* and *K. pneumoniae*. Antibacterial compounds present in various extracts of seaweed might interdict the growth of bacterial pathogens via diverse mechanisms such as inhibition of DNA, RNA and protein synthesis, interference with cell-wall synthesis, lysis of the bacterial membrane and inhibition of metabolic pathways. Antibacterial properties of bioactive compounds significantly influenced the interactions with hydrophobic structures of bacterial strains [82,83,84]. The antibacterial activity of seaweed was due to the presence of fatty acids (Hexadecanoic, 9-octadecenoic, Tetradecanoic and Tetracosenoic acid) [79,80,85,86]. The same result was reported by Raj et al. [87] in which the 500 µg/mL was the minimum inhibitory concentration of *Caulerpa chemnitzia* hexane extract against *S. aureus,* and *K. pneumoniae*.

## 3. Materials and Methods

### 3.1. Collection of Seaweed Caulerpa racemosa 

**Figure 7:**
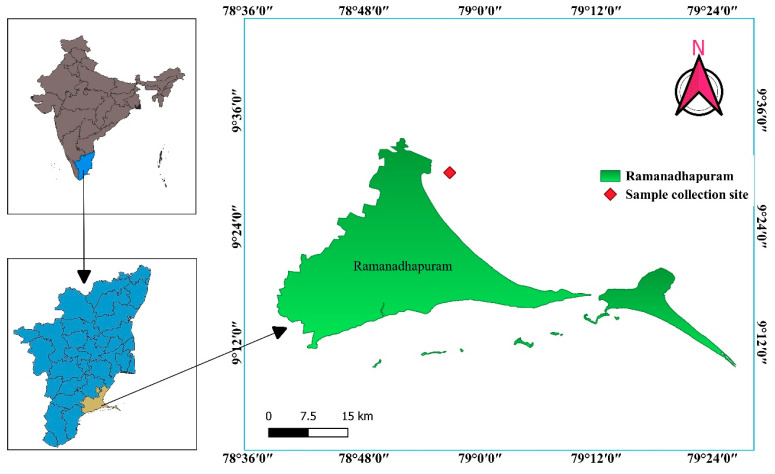
Sample (*Caulerpa racemosa*) collection site mapping.

Seaweed samples were collected from coastal area of Sambai, Ramanadhapuram (9°31′15.3″ N 78°56′08.1″ E) (Figure 7), Tamil Nadu, India. The seaweed was identified by Botanical Survey of India, Southern Regional Station, Tamil Nadu Agricultural University Campus, Coimbatore, India, as *Caulerpa racemosa var. Chemnitzia*. The collected seaweed was washed thoroughly with running faucet water to eliminate surface contaminants. Then, distilled water was used to clean the seaweed, which was then shade dried and cut into small pieces before being ground into fine powder. The powder was stored at −20 °C for further use.

### 3.2. Pigments Determination

An amount of 1 g of crude *Caulerpa racemosa* (CR) powder was homogenized with 10 mL of acetone using a mortar and pestle. The homogenized extract was transferred into the vials then covered with aluminium foil to prevent light penetration and stored at 4 °C for 24 h [88]. Next day, the absorbance was measured spectrometrically (Shimadzu-160A, Japan) at 663, 645, 452.5, 630, 664, 470, 631, 581, 664, 615, 652 and 562 nm.

Chlorophyll a, chlorophyll b, chlorophyll c1+c2, total chlorophyll and carotenoid contents were calculated by using the following formulae according to Arnon’s [89], Dexbury and Yentch [90] and Jensen and Jensen [91]:

Chlorophyll a (mg/g) = 12.7 (A663) – 2.69 (A645)

Chlorophyll b (mg/g) = 22.9 (A645) – 4.68 (A663)

Total chlorophyll (mg/g) = 20.2 (A645) + 8.02 (A663) 

Carotenoids (mg/g) = 4.2 × (A452.5) – (0.0264 × chl. a) + (0.426 × chl. b) 

Chlorophyll c1+c2 (mg/g) = (24.36 × A630) – (3.73 × A664)

### 3.3. Biochemical Constituents Analysis

The proximate composition includes moisture, crude protein, crude fibre, ether extract, total ash and gross energy of CR powder was estimated by using standard AOAC [92] methods.

### 3.4. Preparation of Caulerpa racemosa Solvent Extracts

Based on the polarity, six solvents such as methanol, ethanol (polar), ethyl acetate, acetone (mid polar), petroleum ether and hexane (non-polar) were selected for extraction. Extracts were prepared by maceration method, briefly dissolving 10 g of *C. racemosa* powder in 100 mL of solvent (1:10 *W/V*) [93]. Extracts were kept in a shaker for 24 h at room temperature. Then, the extracts were filtered by Whatman No. 1 filter paper. The filtrate was concentrated with the help of a rotary vacuum evaporator at 40 °C. Desiccated samples were stored at −20 °C until further analysis. For the GC-MS analysis, Soxhlet extraction method was adopted, and the samples were stored at −20 °C until use.

### 3.5. Preliminary Phytochemical Analysis

The prepared CR extracts were investigated to determine the presence of saponins, steroids, terpenoids, phytosterols, flavonoids, tannins, phenol, phenolic flavonoids and alkaloids according to the methods of Sadasivam [94]. The positive results of these tests were considered by observing precipitate formation or any colour change.

#### 3.5.1. Saponins

About 2 mL of distilled water was mixed with 1 mL of CR extracts. The mixture was mixed well for few seconds and allowed to stand for 5 to 10 min. The presence of saponins was determined by foam formation [94]. 

#### 3.5.2. Terpenoids 

Each 1 mL of CR extracts was added to the equal volume of concentrated sulfuric acid (H_2_SO_4_). Terpenoids were detected by the appearance of reddish-brown colour [94].

#### 3.5.3. Steroids

An amount of 0.25 mL of concentrated sulphuric acid (H_2_SO_4_) was added to 0.5 mL of CR extracts along with 1 mL of chloroform. The upper layer turns to yellow, and the lower layer turns to green, fluorescent colour. These colour changes confirm the presence of steroids [94]. 

#### 3.5.4. Phytosterols

An amount of 1 mL of chloroform was added to the equal volume of CR extracts followed by few drops of H_2_SO_4_. This mixture was allowed to stand for few minutes. Presence of golden yellow tint indicates the presence of phytosterol [94]. 

#### 3.5.5. Tannins

An amount of 1 mL of freshly prepared 5% ferric chloride (FeCl_3_) was added to 1 mL of CR extracts. The dark green or greenish black colour formation indicates the presence of tannin [94].

#### 3.5.6. Flavonoids

Few drops of 10% sodium hydroxide (NaOH) were added to 1 mL of CR extracts. The presence of flavonoids was indicated by a brown precipitate [94]. 

#### 3.5.7. Phenol

The phenol was detected by adding few drops of alcoholic FeCl_3_ solution to the 2 mL of CR extract. Formation of bluish colour suggests the presence of phenols [94]. 

#### 3.5.8. Phenolic Flavonoids

Few drops of freshly prepared 10% lead acetate were added to 1 mL of CR extracts. Brown precipitation indicates the presence of phenolic flavonoids [94].

#### 3.5.9. Alkaloids

A total of 1 mL of Mayer’s reagent was added to 1 mL of CR extracts. The existence of alkaloids was confirmed by the formation of a white precipitate [94].

### 3.6. FT-IR Detection

The functional groups present in the different solvent extracts of CR were analyzed by Fourier transform infrared (FT-IR) spectrophotometer (Perkin Elmer, Waltham, MA, USA) by adopting potassium bromide (KBr) pellet method in the spectral range of 4000–500 cm^−1^.

### 3.7. GC-MS Analysis

Shimadzu (QP2020) instrument integrated with a mass spectrometer was used to perform gas chromatography-mass spectrometry (GC-MS) analysis for different solvent extracts of CR. In brief, 100 µL of the filtrate was suspended in 900 µL of respective solvents (ethanol, methanol, acetone, ethyl acetate, hexane and petroleum ether). To eliminate the impurities, the mixture was filtered by a syringe filter (0.25 μM). Then, the filtered samples were injected into Shimadzu (QP2020) GC-MS instrument equipped with 30 m long SH-Rxi-5Sil-MS capillary column (0.25 µm film thickness and 0.25 mm inner diameter) by auto injector in 1:10 split ratio. The inlet temperature program was at 50 °C initially and it was increased gradually (6 °C /min) up to 280 °C. Injector temperature was maintained at 250 °C, pressure at 68.1 kpa and helium was used as a carrier gas with 1.2 mL/min flow rate (linear velocity of 39.7 cm/s). The ionization energy of 70 eV was used to perform ionization in an electron impact mode at 200 °C. The results obtained for CR extracts were compared with the standard mass spectra (NIST 2005 MS collection) libraries. The relative percentage of each compound was determined by calculating the average peak area to total area ratio.

### 3.8. Total Phenolic Content

Folin–Ciocalteu method was used to detect the total phenolic content as described by Salar et al. [95] with slight modifications. The CR extracts of 0.1 mL were added to 0.5 mL of Folin–Ciocalteu reagent. The mixture was kept at 37 °C and incubated for a period of 5 min. Then, 1.5 mL of 7.5% sodium carbonate was added to it and the total volume was made up to 10 mL using distilled water. The absorbance was recorded at 765 nm against blank using Synergy HT Multimode Reader (Bio Tek Instruments, Inc., Winooski, VT, USA). The amount of total phenolic content was calculated using standard gallic acid calibration curve. The results were expressed as mg gallic acid equivalents per gram (mg GAE/g).

### 3.9. Total Tannin Content 

Total tannin content of different solvent extracts of CR was determined by the method of Amorim et al. [96]. Briefly, 0.1 mL of CR extract was diluted with 7.5 mL of distilled water. Then, 0.5 mL of Folin–Ciocalteu reagent was added followed by 1 mL of 35% sodium carbonate. The mixture was mixed well and kept at 25 °C for 30 min. The absorbance was measured at 725 nm. Tannic acid was used as standard, and the results were expressed as mg tannic acid equivalents per gram (mg TAE/g) using the calibration curve of tannic acid.

### 3.10. Total Flavonoid Content 

Aluminium chloride (AlCl_3_) colorimetric assay of Lamaison and Carnart [97] was adopted to determine the total flavonoid content of CR solvent extracts. In brief, 0.2 mL of CR extracts were added to a test tube containing 4.8 mL of distilled water. Then, 0.3 mL of 5% sodium nitrite (NaNO_2_) was added and mixed well using a vortex mixer. After 5 min, 0.3 mL of 10% AlCl_3_ ∙6H_2_O was added, followed by the addition of 2 mL of 1M NaOH and the total volume was made up to 10 mL with distilled water. The absorbance was measured at 414 nm. Quercetin was used as standard, and the total flavonoid content was expressed as mg quercetin equivalents per gram (mg QE/g) using the calibration curve of quercetin.

### 3.11. In Vitro Antioxidant Activity 

#### 3.11.1. DPPH Radical-Scavenging Activity

DPPH (2, 2-diphenyl-1-picryl-hydrazile) activity was estimated according to the method of Brand-Williams et al. [98]. The reaction was performed in a 96-well microtiter plate containing 100 µL of different concentrations (20 to 100 µg/mL) of CR extracts. Then, 100 µL of 2mM DPPH solution was added to each well. The reaction mixture was incubated at room temperature in dark conditions for 30 min. The coloration from violet to yellow indicates free radical scavenging activity by the compounds present in the CR extracts. The change in absorbance was read at 517 nm using HT Multimode Reader (Bio Tek Instruments, Inc., Winooski, VT, USA). Vitamin C (Ascorbic acid) was used as standard. The following formula Equation (1) was used to calculate the percentage of CR extracts’ radical scavenging ability,

(1) $$\%\;of\;DPPH\;scavenging=\frac{Ab-As}{Ab}\times 100$$

where *Ab*—absorbance value of blank and *As*—absorbance value of sample.

#### 3.11.2. ABTS Radical Scavenging Activity

ABTS [2,2′-azino-bis (3-ethylbenzothiazoline-6-sulphonic acid)] radical scavenging activity was determined by cation decolorization assay with slight modifications in Arumugam et al. [99] to analyze the antioxidant potential of various solvent extracts of CR. The ABTS stock solution was prepared by mixing equal volumes of 7 mM ABTS and 140 mM of potassium persulfate solution and allowed them to react in dark conditions at 25 °C for 12–16 h before use. The working solution was prepared by diluting the stock using 50% ethanol to obtain an absorbance of 0.7 ± 0.02 at 734 nm using HT Multimode Reader (Bio Tek Instruments, Inc., Winooski, VT, USA). Subsequently, 200 µL of ABTS solution was added to 100 µL of various concentrations of CR extracts in a 96-well microtiter plate. The mixture was incubated in a dark condition for 10 min and then, the absorbance was read at 734 nm. Ascorbic acid was used as standard. The percentage of inhibition was calculated using Equation (1).

### 3.12. Anti-Bacterial Activity

The antibacterial potential of various solvent extracts of CR was studied against aquatic Gram-negative pathogens such as *Aeromonas hydrophila*, *Aeromonas salmonicida*, *Aeromonas veronii*, *Klebsiella pneumoniae*, *Pseudomonas aeruginosa* and Gram-positive pathogen *Staphylococcus aureus*, by agar well diffusion method according to Logaranjan et al. [100] with slight modifications. Briefly, the bacteria were pre-cultured overnight at 37 °C. The culture strain was adjusted to obtain a final concentration of 1 × 10^8^ cells/mL using 0.5 McFarland standards and inoculated in triplicates on Muller-Hinton agar plates using a sterile cotton swab. Then, a well was created using corkborer in the inoculated plates. The sample extracts were resuspended in Dimethyl sulfoxide (DMSO) with a concentration of 1 mg/mL to reduce the evaporation rate. Different concentrations (50, 100, 150 and 200 µg/mL) of CR extracts were added to the wells. DMSO and streptomycin (1 mg/mL) were used as a negative and positive control, respectively. Then, the plates were incubated at 37 °C overnight. Finally, the antibacterial activity was determined by measuring the zone of inhibition (mm) formed around the wells.

### 3.13. Minimum Inhibitory Concentration (MIC) Determination

Resazurin-based 96 well microtiter plate method of Chakansin et al. [101] was adopted to determine the MIC of various extracts of CR with slight modifications. In brief, 100 µL of nutrient broth was added to the sterile 96-well microtiter plate. First row of the plate acted as negative control (nutrient broth). Second row of the plate acted as positive control (streptomycin). Serial dilutions were made from third row of the plate containing 100 µL of CR extracts which was resuspended in DMSO. Finally, 50 µL of bacterial suspension was added to all the wells resulting in a final concentration of 1 × 10^7^ CFU/mL. To avoid dehydration, the plate was loosely wrapped with aluminium foil, and it was incubated at 37 °C for 24 h. After incubation, 20 µL of resazurin indicator solution was added to all the wells. Then, the plate was incubated again for 2–4 h at 37 °C. The results were examined visually. The colour change from purple to pink indicates the reduction in resazurin by bacteria. The experiment was performed in triplicates and the lowest concentration that prevented the colour change was considered as the MIC value.

### 3.14. Statistical Analyses

Experiments were performed in triplicates and the results were presented as mean ± standard deviation. The data were analyzed by applying two-way ANOVA with Tukey’s multiple comparisons test using GraphPad Prism version 8 (GraphPad Software, Inc., San Diego, CA, USA). The data are presented in the form of descriptive statistics through tables and graphs. *, **, ***, and **** indicate *p*-values of, respectively, ≤0.05, ≤0.01, ≤0.001 and ≤0.0001.

## 4. Conclusions

In this study, our results demonstrated that the various solvent extracts of *C. racemosa* exhibited significant in vitro properties. Among all the extracts evaluated, the methanol extract showed better results than other solvent extracts both in antioxidant and antibacterial activities. The levels of tannin and flavonoid content in the methanol extract might be responsible for its increased biological activities. The GC-MS analysis revealed the presence of pentadecane, 1-heptadecene, tridecanoic acid, methyl ester, 2-aminophenol and hexadecamethyl compounds in the solvent extracts of *C. racemosa* endowed with potential antioxidant and antibacterial properties, which are responsible for the wider production of novel drugs that could be facilitated to treat or prevent infectious diseases for humans and animals. In futuristic strategies, the marine seaweed would be utilized as a sustainable novel natural drug development approach for therapeutics, nutraceutical and pharmaceutical large-scale industrial applications. However, extensive investigations should be warranted to exploit the action mechanisms of the *C. racemosa* extracts and its bioactive compounds and evaluate the effects in biological systems in vivo using experimental animal models. 

## Figures and Tables

**Table 1:** Biochemical constituents’ analysis of *Caulerpa racemosa*.

Biochemical Constituents	*Caulerpa racemosa*
Moisture	7.04%
Crude protein	12.64%
Crude fibre	2.85%
Ether extract	1.80%
Total ash	48.41%
Nitrogen free extract	27.26%
Gross energy	2089 Kcal/kg

**Table 2:** Preliminary phytochemical analysis of various extracts of *Caulerpa racemosa*. “+”—indicates presence of phytochemicals. “–”—indicates the absence of phytochemicals.

S. No	Test	Methanol	Ethanol	Acetone	Ethyl Acetate	Petroleum Ether	Hexane
1.	Saponins	+	+	–	–	–	–
2.	Terpenoids	+	+	+	+	–	+
3.	Steroids	+	+	+	+	–	–
4.	Phytosterol	+	+	+	+	–	–
5.	Tannins	+	+	+	+	+	+
6.	Flavonoids	+	+	+	+	–	–
7.	Phenol	+	+	+	–	–	–
8.	Phenolic flavonoids	+	+	+	–	–	–
9.	Alkaloids	+	+	–	–	–	–

**Table 3:** GC–MS analysis of various extracts of *Caulerpa racemosa*.

Extract	Compound Name	Molecular Formula	Molecular Weight	Area %
Methanol	Oxalic acid, allyl ethyl ester	C_8_H_10_O_4_	170	0.11
	3-Butynoic acid	C_4_H_4_O_2_	84	0.03
	3-Hexadecene	C_16_H_32_	224.42	0.8
	Phthalic acid	C_8_H_6_O_4_	166.14	0.23
	Dodecane	C_12_H_26_	170.33	2.16
	3-Octadecene, (E)-	C_18_H_36_	252.5	3.3
	Pentadecane	C15H_32_	212.41	12.99
	Heptadecane, 7-methyl-	C_18_H_38_	254.5	0.48
	Carbonic acid, decyl vinyl ester	C_13_H_24_O	228.33	0.15
	1-Heptadecene	C_17_H_34_	238.5	32.37
	1-Decene, 3,3,4-trimethyl-	C_13_H_26_	160.21	0.27
	Pentadecane	C_15_H_32_	212.42	2.72
	Neophytadiene	C_20_H_38_	278.5	0.60
	2-Tridecenal, (E)-	C_13_H_24_O	196.33	0.39
	9-Heptadecanone	C_17_H_34_O	254.5	1.11
	Tetradecane	C_14_H_30_	198.39	0.28
	9-Octadecenoic acid (Z)- methyl ester	C_19_H_36_O_2_	296.5	0.22
	Tridecanoic acid, methyl ester	C_14_H_28_O_2_	228.37	8.53
	1,1-Diisobutoxy-butane	C_12_H_26_O_2_	202.33	0.36
	Nonane, 3,7-dimethyl-	C_11_H_24_	156.31	0.18
	1-Dodecene, 2-ethyl-	C_12_H_24_	168.32	0.29
	8,11,14-Eicosatrienoic acid, methyl ester,	C_21_H_36_O_2_	320.5	0.60
	11,14-Eicosadienoic acid, methyl ester	C_21_H_38_O_2_	322.5	0.43
	7-Hexadecenoic acid, methyl ester, (Z)-	C_17_H_32_O_2_	268.4	0.24
	Tetracosanoic acid, methyl ester	C_25_H_50_O_2_	382.7	0.26
	1-Nonadecene	C_19_H_38_	266.5	0.80
	2-Aminophenol, 2TBDMS derivative	C_18_H_35_NOSi_2_	337.6476	12.77
	Heneicosane	C_21_H_44_	296.57	6.37
	Octasiloxane, 1,1,3,3,5,5,7,7,9,9,11,11 13,13,15,15- Hexadecamethyl(Alpha reductase inhibitor, 5-HT inhibitor)	C_16_H_50_O_7_Si_8_	578	27.47
Ethanol	3-Hexadecene	C_16_H_32_	224.42	1.92
	Acetic acid			13.57
	2-(Benzyloxy)ethanamine	C_9_H_13_NO	151	13.27
	Propiolactone	C_3_H_4_O_2_	72	4.98
	N-(4-Tolylsulfonyl)azetidin-3-one	C_10_H_11_NO_3_S	225	10.38
	1H-Tetrazole	CH_2_N_4_	70	2.87
	N-Methylene-2-phenylethanamine	C_9_H_11_N	133	1.43
	Butanenitrile	C_4_H_7_N	69.11	2.90
	Hexadecane	C_16_H_34_	226	1.17
	Neophytadiene	C_20_H_38_	278	5.63
	3,7,11,15-Tetramethyl-2-hexadecen-1-ol	C_20_H_40_O	296.5	11.06
	Hexadecanoic acid, ethyl ester	C_18_H_36_O_2_	284	2.10
Acetone	Propanoic acid	C_3_H_6_O_2_	74.08	6.15
	2-Pentanone, 4-hydroxy-4-methyl	C_18_H_20_O_2_	116.16	25.88
	Acetic acid, hydroxy-, methyl ester (methyl glycolate)	C_3_H_6_O_3_	90.08	0.90
	(3S,4S)-3,4-Bis(methoxymethoxy)pyrrolidine	C_8_H_17_NO_4_	191	0.34
	Oxalic acid, diallyl ester	C_8_H_10_O_4_	170.16	1.28
	Butanenitrile	C_4_H_7_N	69.11	1.84
	Heptadecane	C_17_H_36_	240.471	7.57
	3,7,11,15-Tetramethyl-2-hexadecen-1-ol	C_20_H_40_O	296.5	5.78
Ethyl acetate	1H-Tetrazole	CH_2_N_4_	70	8.20
	Propiolactone	C_3_H_4_O_2_	72	4.42
	2-Butanol, 4-chloro-3-methyl-	C_5_H_11_ClO	122.59	4.08
	Hexahydro-1,3,5-trinitroso-1,3,5-triazine	C_3_H_6_N_6_O_3_	174	7.45
	2-Butanone, 3-hydroxy	C_4_H_8_O	88.11	3.19
	2-Benzyloxyethylamine	C_19_H_13_NO	271	10.05
	Propanoic acid	C_3_H_6_O_2_	74.08	7.76
	1-Tridecene	C_13_H_26_	182	6.96
	1-Heptadecene	C_17_H_34_	238.5	5.45
Petroleum ether	Propiolic acid	C_3_H_2_O_2_	70.05	0.87
	2-Pentanone, 5-hydroxy-	C_5_H_10_O_2_	102	19.53
	1H-Tetrazole	CH_2_N_4_	70	5.87
	2-Tetradecanol	C_14_H_30_O	214	1.06
	Tricosane	C_23_H_48_	324	1.27
	Hexanoic acid	C_6_H_12_O_2_	116.15	3.25
	Isopropyl myristate	C_17_H_34_O_2_	270.45	4.16
	Pentadecanoic acid, methyl ester	C_17_H_34_O_2_	270	3.58
	Hexanedioic acid, bis(2-ethylhexyl) ester	C_22_H_42_O_4_	370.6	2.35
Hexane	Cyclopentane, 1-acetyl-1,2-epoxy	C_7_H_10_O_2_	126	54.36
	N,N′,N″-Trinitro-1,3,5-triazacycloheptane	C_4_H_8_N_6_O_6_	36	6.89
	1H-Tetrazole	CH_2_N_4_	70	6.66
	Propiolactone	C_3_H_4_O_2_	72	2.12
	Butanenitrile	C_4_H_7_N	69	0.64
	Tricosane	C_23_H_48_	324	1.77
	Pentadecane	C_15_H_32_	212	3.50

**Table 4:** IC_50_ values of *Caulerpa racemosa* extracts of DPPH & ABTS radical scavenging activity.

Extracts of *Caulerpa racemosa*	DPPH Assay (µg/mL)	ABTS Assay (µg/mL)
Vitamin C (standard)	36.79	32.06
Methanol	86.33	54.51
Ethanol	104.46	75.10
Acetone	102.52	73.64
Ethyl acetate	111.59	74.41
Petroleum ether	124.41	69.92
Hexane	173.21	76.28

**Table 5:** Antibacterial activity and MIC of the various extracts of *Caulerpa racemosa* against tested microorganisms.

		Zone of Inhibition (mm)					
Extract	Bacterial Strain	Control (Streptomycin)	50µg/mL	100 µg/mL	150 µg/mL	200 µg/mL	MIC µg/mL
Methanol	*Aeromonas hydrophila*	25.5 ± 2.12	-	15.5 ± 0.72 **	17.5 ± 2.12 **	21.5 ± 2.12 *	100
	*Aeromonas veronii*	29 ± 1.41	-	20 ± 2.82 *	24 ± 2.83 *	27 ± 0.71 *	100
	*Aeromonas salmonicida*	26.5 ± 0.70	-	16 ± 1.41 **	17.5 ± 2.12 **	23.5 ± 0.71 *	100
	*Pseudomonas aeruginosa*	29 ± 1.41	-	11.5 ± 2.12 **	14 ± 0.70 **	19.5 ± 2.12 *	200
	*Staphylococcus aureus*	26.5 ± 0.70	-	12.25 ± 1.06 **	15 ± 1.41 **	17.5 ± 0.71 **	200
	*Klebsiella pneumoniae*	26 ± 2.83	-	12 ± 1.41 **	14 ± 1.41 **	17.75 ± 1.06 **	200
Ethanol	*Aeromonas hydrophila*	30 ± 2.83	-	12.5 ± 0.71 **	16 ± 1.41 **	19.5 ± 0.71 *	100
	*Aeromonas veronii*	29.5 ± 2.12	-	16.5 ± 2.12 **	19 ± 2.83 *	25 ± 0.35 *	100
	*Aeromonas salmonicida*	32.75 ± 0.35	-	14.5 ± 2.12 **	17.5 ± 0.71 **	21.25 ± 0.35 *	100
	*Pseudomonas aeruginosa*	24.5 ± 0.71	-	12 ± 1.41 **	14 ± 1.41 **	16.5 ± 0.71 **	200
	*Staphylococcus aureus*	24.5 ± 0.71	-	10.75 ± 0.35 **	11.5 ± 0.71 **	13.5 ± 0.71 **	200
	*Klebsiella pneumoniae*	29.5 ± 0.71	-	-	11.25 ± 1.06 **	13.5 ± 2.12 **	200
Acetone	*Aeromonas hydrophila*	28.5 ± 2.12	-	11 ± 1.14 **	13.5 ± 0.71 **	16 ± 1.41 **	100
	*Aeromonas veronii*	31 ± 1.41	-	14.5 ± 2.12 **	18 ± 2.83 **	21.5 ± 0.71 *	100
	*Aeromonas salmonicida*	27.5 ± 2.12	-	15.5 ± 0.71 **	16 ± 1.41 **	18 ± 1.41 **	100
	*Pseudomonas aeruginosa*	29 ± 1.41	-	-	10.5 ± 0.71 **	11.5 ± 0.71 **	200
	*Staphylococcus aureus*	25.5 ± 2.12	-	10.5 ± 0.71 **	11.5 ± 0.71 **	11.75 ± 1.06 **	200
	*Klebsiella pneumoniae*	28.5 ± 0.71	-	-	10.5 ± 0.71 **	13.5 ± 0.71 **	200
Ethyl acetate	*Aeromonas hydrophila*	29 ± 0.71	-	11 ± 1.41 **	12 ± 2.82 **	15.5 ± 0.71 **	200
	*Aeromonas veronii*	30.5 ± 0.71	-	12 ± 2.83 **	15.5 ± 2.12 **	20 ± 1.41 *	100
	*Aeromonas salmonicida*	29.5 ± 2.12	-	12.5 ± 0.71 **	13.5 ± 2.12 **	15.5 ± 0.71 **	100
	*Pseudomonas aeruginosa*	29 ± 1.14	-	-	-	10.5 ± 0.71 **	200
	*Staphylococcus aureus*	27.25 ± 0.35	-	11 ± 0.35 **	11 ± 0.35 **	11.5 ± 0.71 **	200
	*Klebsiella pneumoniae*	26 ± 2.83	-	-	11.5 ± 0.71 **	11 ± 1.41 **	400
Petroleum ether	*Aeromonas hydrophila*	26.5 ± 0.71	-	-	-	12 ± 1.41 **	400
	*Aeromonas veronii*	28.5 ± 0.71	-	11.5 ± 2.12 **	11.5 ± 0.71 **	14.25 ± 1.06 **	200
	*Aeromonas salmonicida*	26.5 ± 0.71	-	11.5 ± 0.71 **	10.75 ± 1.06 **	11.5 ± 0.71 **	200
	*Pseudomonas aeruginosa*	29.5 ± 2.12	-	-	11 ± 0.71 **	12 ± 1.41 **	400
	*Staphylococcus aureus*	28.5 ± 2.12	-	-	10.5 ± 0.71 **	12.5 ± 0.71 **	400
	*Klebsiella pneumoniae*	27 ± 1.41	-	-	-	11.5 ± 2.12 **	400
Hexane	*Aeromonas hydrophila*	27.5 ± 0.71	-	-	10.5 ± 0.71 **	12 ± 1.41 **	400
	*Aeromonas veronii*	30 ± 0.71	-	10.5 ± 0.71 **	13.5 ± 0.71 **	16 ± 1.41 **	200
	*Aeromonas salmonicida*	32 ± 1.41	-	10.5 ± 0.71 **	10.5 ± 0.71 **	12 ± 1.41 **	400
	*Pseudomonas aeruginosa*	29 ± 1.41	-	-	-	13.5 ± 0.70 **	400
	*Staphylococcus aureus*	26 ± 1.41	-	-	11 ± 1.14 **	12 ± 2.82 **	400
	*Klebsiella pneumoniae*	27.5 ± 0.71	-	-	-	12.5 ± 2.12 **	200

Each result represents the mean±standard deviation (n = 3), and asterisks indicate significant differences between the control and different concentrations of *Caulerpa racemosa* extracts. “-” indicates no activity.

## Data Availability

All data generated or analyzed during this study are included in this published article.
